# The Impact of Neighbourhood Deprivation on Embryonic Growth Trajectories: Rotterdam Periconception Cohort

**DOI:** 10.3390/jcm8111913

**Published:** 2019-11-08

**Authors:** Dionne V. Gootjes, Maria P. H. Koster, Sten P. Willemsen, Anton H. J. Koning, Eric A. P. Steegers, Régine P. M. Steegers-Theunissen

**Affiliations:** 1 Department of Obstetrics and Gynaecology, Erasmus MC, University Medical Centre Rotterdam, Rotterdam 3015 GD, The Netherlandsm.p.h.koster@erasmusmc.nl (M.P.H.K.); s.willemsen@erasmusmc.nl (S.P.W.); e.a.p.steegers@erasmusmc.nl (E.A.P.S.); 2 Department of Biostatistics, Erasmus MC, University Medical Centre Rotterdam, Rotterdam 3015 GD, The Netherlands; 3 Department of Pathology, Erasmus MC, University Medical Centre Rotterdam, Rotterdam 3015 GD, The Netherlands; a.koning@erasmusmc.nl

**Keywords:** social class, pregnancy, geographic origin, residence characteristics, social determinants of health

## Abstract

Background: Neighbourhood deprivation is a risk factor for impaired health and adverse pregnancy outcomes. We investigated whether living in a deprived neighbourhood is associated with embryonic growth. Methods: From the Predict cohort, we studied 566 women who underwent repeated first trimester ultrasound examinations. Crown rump length (CRL; *n* = 1707) and embryonic volume (EV; *n* = 1462) were measured using three-dimensional techniques. Neighbourhood deprivation was assessed using the neighbourhood status scores (NSS) of the Dutch Social Cultural Planning office. A high NSS represents a non-deprived neighbourhood. Associations between the NSS and embryonic growth were investigated using linear mixed models. Adjustment was performed for individual-level factors: maternal age, geographic origin, educational level, BMI, folic acid supplement use, fruit and vegetable intake, alcohol use and smoking habits. Results: The NSS was negatively associated with embryonic growth: a higher score (a less deprived neighbourhood) was associated with a smaller CRL and EV; adjusted β: −0.025 (95% CI −0.046, −0.003) and adjusted β: −0.015 (95% CI −0.026, −0.003). At 11 weeks of pregnancy, we observed a 0.55 cm^3^ smaller EV (7.65 cm^3^ vs. 7.10 cm^3^) and 1.08 mm smaller CRL (43.14 mm vs. 42.06 mm) in the highest compared to the lowest category. Conclusion: In deprived neighbourhoods, embryos are larger than in non-deprived neighbourhoods.

## 1. Introduction

Residential neighbourhood environment is acknowledged as an important factor affecting health and reproductive outcomes of the population [1,2,3]. Living in a deprived neighbourhood has been shown to be associated with adverse pregnancy outcomes such as preterm birth, small-for-gestational age (SGA) and perinatal mortality [4,5]. Unravelling the underlying mechanisms through which living in a deprived neighbourhood impacts health and pregnancy course and outcome remains challenging. It is suggested that the accumulation of risk factors within an individual such as unhealthy nutrition, substance abuse and smoking results in health inequalities between neighbourhoods [6]. Therefore, neighbourhood deprivation is often used as a composite determinant of health. However, an isolated role of neighbourhood deprivation has also been postulated; living in a deprived neighbourhood is considered a self-contained risk factor for impaired physical and mental health and pregnancy course and outcomes [5,7].

It is known that the course of pregnancy largely originates in the periconception period, which covers the 14 weeks before and 10 weeks after conception [8]. This period is critical since the developing gametes, embryo and placenta are susceptible to changes due to maternal conditions and environmental factors, including nutrition and lifestyle factors. For instance, a diet characterised by high intakes of vegetable oil and fish is associated with improved embryonic development [9,10]. Moreover, the absence of folic acid supplement intake in this period is associated with congenital malformations and even impaired embryonic growth [11,12,13]. We hypothesize that in this vulnerable period, the developing embryo is also susceptible to environmental factors such as neighbourhood deprivation. A commonly used parameter of embryonic size is the crown–rump length (CRL), traditionally obtained at the end of the first trimester of pregnancy using two-dimensional ultrasound techniques. The introduction of three-dimensional ultrasound (3D-US) enabled embryonic growth assessment in a highly precise manner. At our institution, 3D-US datasets can be projected in the Barco I-Space, a system that uses stereo projection on three screens and the floor to create (embryonic) holograms. The system allows full depth perception and interaction with the projected hologram and enables more precise CRL measurements [14]. Besides, it gives the opportunity to assess the volume of an embryo; the embryonic volume (EV). This volume is measured by a segmentation algorithm, which allows instant and semi-automated volume measurements [15]. This volume measurement technique has been previously described elsewhere and validated in a number of studies [14,16]. The EV is as a more accurate measure of embryonic size [15,17].

Since embryonic growth is associated with second and third trimester growth and pregnancy outcomes, impaired growth in these trimesters and adverse pregnancy outcomes might be preceded by an already impaired development from as early as the first trimester of pregnancy onwards [12]. This study aims to elucidate whether the previously demonstrated negative effect of living in a deprived neighbourhood on course and outcome of pregnancy is already detectable in the growth trajectory of the embryo during the first trimester of pregnancy. This might affirm the need to take the residential environment into account when evaluating individual’s risk factors for impaired intrauterine growth. Moreover, this could provide the possibility for early detection of intrauterine growth retardation. Then, early interventions can be implemented, such as those aimed to improve nutrition and lifestyle behaviours, which are demonstrated to be able to prevent adverse outcomes of impaired intrauterine growth [18,19].

From this background, we investigated the associations between neighbourhood deprivation and embryonic growth trajectories.

## 2. Materials and Methods

### 2.1. Cohort Selection

**Figure 1:**
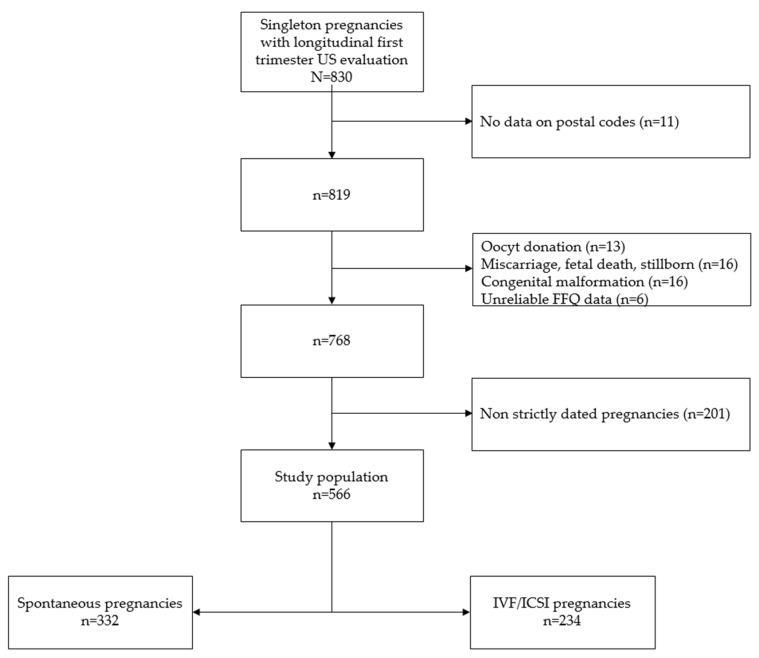
Flowchart of the study population. US: ultrasound, FFQ: food frequency questionnaire, IVF: in vitro fertilization, ICSI: intracytoplasmic sperm injection.

This study was performed using data from the ongoing Rotterdam Periconception Cohort (Predict study), an ongoing prospective tertiary hospital-based study, embedded in the outpatient clinic of the department of Obstetrics and Gynaecology of the Erasmus MC, University Medical Centre Rotterdam, the Netherlands. A detailed description of the cohort has previously been published [20]. In short, women with a singleton pregnancy and of at least 18 years of age were eligible for participation and were recruited prior to 10 weeks of gestation between November 2010 and November 2016. Women without information on the area of residency at the time of study enrolment, pregnancies conceived after oocyte donation, pregnancies complicated by spontaneous miscarriages, intrauterine deaths, perinatal deaths or congenital anomalies were excluded from analysis. Moreover, when either ultrasound or maternal nutrition data was missing or unreliable (i.e., a reported total energy intake of <500 kcal/day), pregnancies were also excluded (Figure 1).

### 2.2. Data Collection

Participating pregnant women completed a self-administered questionnaire at enrolment providing details on age, geographic origin, educational level, medical and obstetrical history and lifestyle behaviours (i.e., folic acid supplement use, smoking and alcohol consumption). Anthropometric measurements (height and weight) were measured by a research nurse at the first study visit and used to calculate body mass index (BMI). Furthermore, a food frequency questionnaire (FFQ) was completed, by which detailed information about vegetable and fruit intake and total their energy intake was collected [21]. Adequate nutrition and lifestyle behaviours were defined as a vegetable intake of ≥200 g/day, a fruit intake of ≥2 pieces/day (equivalent to/more than 200 g), initiation of folic acid supplement use (400 µg/day) before pregnancy, no smoking and no alcohol consumption [12,22,23,24,25].

### 2.3. Pregnancy Dating

Since gestational age (GA) is the most important determinant of embryonic growth, accurate pregnancy dating is vital. In clinical practice, pregnancy dating is mostly based on first trimester CRL measurement. However, since CRL is one of the outcome measures of this study, we did not calculate GA based on the CRL. In spontaneously conceived pregnancies, including those derived after intra-uterine insemination (IUI) and hormone therapy, GA was calculated based on the first day of the last menstrual period (LMP). Therefore, of the spontaneously conceived pregnancies, only women with a known LMP and regular menstrual cycle (between 25 and 35 days) were included. In pregnancies conceived after in vitro fertilization (IVF) treatment with or without intracytoplasmic sperm injection (ICSI), GA was calculated based on the date of oocyte retrieval plus 14 days. In pregnancies conceived after the transfer of cryopreserved embryos, GA was calculated from the day of embryo transfer plus 19 days [20,26].

### 2.4. Exposure

The neighbourhood status score (NSS), as assessed by The Netherlands Institute for Social Research, was used as a composite determinant of the deprivation status of a participant’s neighbourhood [27]. These scores are determined for all 4-digit zip codes in the Netherlands that have more than 100 households per region, and are based on four elements: the average income, the number of non-employed residents, the number of lower educated residents and the number of households with a low income in that particular neighbourhood. The scores are standardized according to a normal distribution; the average over all the years is 0, with a standard deviation of 1. A high NSS represents a non-deprived status, whereas a low NSS represents a deprived status of the neighbourhood. The NSS is calculated every four years and for this study, the NSS of the year 2014 was used to determine the classification of the neighbourhood of the participants. In that year, the interquartile range (IQR) of the NSS in the Netherlands was −0.57 to 0.71. A total of 193 neighbourhoods included in the study, based on the residential address at the moment of inclusion in the study (early in pregnancy). To illustrate growth trajectories of the embryos of women who live in neighbourhoods with different NSS, three groups were created based on their scores: a low NSS (a score up to −1), an intermediate NSS (a score between −1 and 1) and a high NSS (a score higher than 1).

### 2.5. Outcomes

Women underwent longitudinal transvaginal 3D-US scans at 7, 9 and 11 weeks GA with a 6–12 MHz transvaginal probe using GE Voluson E8 equipment and 4D View software (General Electrics Medical Systems, Zipf, Austria). The 3D-US datasets were transferred to the Barco I-Space (Barco N.V., Kortrijk, Belgium) to create a virtual reality hologram which allows depth perception and interaction with the hologram. Trained researchers performed offline CRL and EV measurements. The feasibility and reliability of these measurements have previously been described, and excellent inter- and intra-observer agreement was established [14,15]. CRL measurements were performed three times and the average of these measurements was used in the analysis. EV measurements were performed once in the same image that was used for the CRL measurements.

### 2.6. Statistical Analysis

Maternal characteristics are presented in Table 1. The median and interquartile range is presented for data with a skewed distribution. Baseline characteristics were compared between women in and excluded from analysis (Table S1). Differences were tested with Mann-Whitney U test for variables with a skewed distribution and Chi square tests for categorical variables.

Associations between the NSS and embryonic growth were assessed through linear mixed model analysis. In these models, the NSS was used as a continuous measure. Linear mixed models take the dependent serial measurements of CRL and EV of the same pregnancy into account. Potential confounders were identified based on significant associations between maternal characteristics, nutrition and lifestyle behaviours, and NSS (Table 2), as well as the previously studied associations between covariates and embryonic growth in literature. In model 1 we adjusted for GA at the time of ultrasound. In model 2, we additionally adjusted for maternal age (continuous), gestational age (continuous), geographic origin (Western vs. non-Western), educational level (low, intermediate, high), BMI (continuous), folic acid supplement use (adequate vs. inadequate), fruit and vegetable intake (adequate vs. inadequate), alcohol use (adequate vs. inadequate), smoking habits (adequate vs. inadequate) and mode of conception (IVF/ICSI vs. spontaneous).

Pregnancy dating is performed in different ways between the IVF/ICSI pregnancies and spontaneous pregnancies in this study population. Moreover, IVF/ICSI treatment is associated with increased risks of preterm birth, foetal growth restriction, a child small for gestational age (SGA) and low birthweight and therefore potentially with embryonic growth [28,29,30]. Therefore, sensitivity analyses were executed, stratified according to the mode of conception (IVF/ICSI pregnancies versus spontaneous pregnancies: Table S2).

Approximate conditionally normal distributions of the responses and linear associations with GA were obtained by square root transformation of CRL and cube root transformation of EV. Retransformation of the CRL and EV to the original scale allowed us to calculate the absolute and relative differences of the CRL (in mm) and EV (in cm^3^) between the highest and lowest NSS group, at 7 and 11 weeks of pregnancy. Statistical analyses were performed using the Statistical Package for the Social Sciences version 23.0 for Windows (SPSS Inc, Chicago, IL, USA). *p*-values below 0.05 were considered statistically significant.

### 2.7. Ethics Approval

This study was conducted according to the guidelines laid down in the Declaration of Helsinki. The protocol was approved by the Medical Ethical and Institutional Review Board of the Erasmus MC, University Medical Centre in Rotterdam (MEC-2004-277). All participants provided written informed consent.

## 3. Results

### 3.1. Baseline Characteristics

From a total of the 830 pregnancies with serial ultrasound evaluation in the Rotterdam Periconception cohort, 566 pregnancies were included in the analyses (Figure 1). Compared to the women excluded from the analysis, the included women had a higher age (32 years vs. 31 years), a more adequate intake of fruit and vegetables (55.2% vs. 42.0% and 34.8 vs. 23.9%, respectively), and the included women more frequently used folic acid supplements (82.6% vs. 67.4%) (Table S1). Of the 566 included women, 332 (58.7%) conceived spontaneously and 234 (41.3%) conceived after IVF or ICSI treatment. At the moment of inclusion, the median age of the total study population was 32 years (IQR 29–36) and the median BMI was 24.4 kg/m^2^ (IQR 21.9–28.2). Women living in a neighbourhood with a low NSS (NSS < −1; deprived neighbourhood) were less often of Western geographic origin (73.6% vs. 92.9%), were less often highly educated (58.9% vs. 77.7%) and had a higher median BMI (25.3 kg/m^2^ vs. 23.3 kg/m^2^) compared to women living in a neighbourhood with a high NSS (NSS > 1; non-deprived neighbourhood) (Table 1).

In the IVF/ICSI group, women were older (33 (IQR 30–36) vs. 32 (IQR 29–35) years; *p* < 0.001), more often nulliparous (64.6% vs. 34.0%; *p* < 0.001), less often highly educated (highly educated, 50.9% vs. 62.9%; *p* < 0.001) and more often used folic acid supplements (97.3% vs. 72.7%; *p* < 0.001) compared to the group of women who conceived spontaneously (Table S2). The NSS of women in the IVF/ICSI group was higher compared to women in the spontaneously conceived group (0.08 (IQR −0.84–0.77) vs. –0.04 (IQR −1.36–0.77); *p* < 0.001), indicating that these women more often live in neighbourhoods with the highest NSS; the non-deprived neighbourhoods.

Women living in neighbourhoods with a higher NSS were less likely to have a non-Western geographic origin β: −1.30 (95% CI −1.49, −1.10) and to have an inadequate folic acid supplement intake β: −0.47 (95% CI −0.64, −0.29). They were more likely to be higher educated β: 0.29 (95% CI 0.15, 0.43), to have a lower BMI β: −0.05 (95% CI −0.06, −0.04) and to have conceived after IVF/ICSI treatment β: 0.37 (95% CI 0.25, 0.50) (Table 2).

### 3.2. Embryonic Growth Trajectories

In the crude models, NSS was not associated with the embryonic growth trajectories (CRL: crude β: −0.015 (95% CI −0.034, 0.003) and EV: β: −0.008 (95% CI −0.018, 0.001)) (Table 3). However, in the adjusted models, NSS was significantly negative associated with the serial embryonic growth trajectories CRL and EV (adjusted β: −0.025 (95% CI −0.046, −0.003) and adjusted β: −0.015 (95% CI −0.026, −0.003), respectively). In the stratified analyses for mode of conception, effect sizes were comparable, in both spontaneously conceived and IVF/ICSI pregnancies. The negative association between NSS and CRL and EV was more pronounced in spontaneously conceived pregnancies (CRL, adjusted β: −0.031 (95% CI −0.059, −0.002) and adjusted β: −0.020 (95% CI −0.034, −0.006)) (Table S3).

Retransformation to the original scale demonstrated that the CRL of embryos from women who live in a neighbourhood with a high NSS (i.e., non-deprived) was on average 0.49 mm smaller (relative difference 5.49%; 8.90 mm vs. 8.42 mm) at 7 weeks gestation and 1.08 mm smaller (relative difference 2.51%; 43.14 mm vs. 42.06 mm) at 11 weeks gestation compared to embryos from women who live in a neighbourhood with a low NSS (i.e., deprived). The EV of embryos from women who live in neighbourhoods with a high NSS was on average 0.02 cm^3^ smaller (relative difference 33.77%: 0.05 cm^3^ vs. 0.03 cm^3^) at 7 weeks gestation and 0.55 cm^3^ smaller (relative difference 7.13%: 7.65 cm^3^ vs. 7.10 cm^3^) at 11 weeks of gestation compared to embryos from women who live in neighbourhoods with a low NSS (Figure S1).

## 4. Discussion

### 4.1. Main Findings

We found that increasing neighbourhood deprivation is associated with larger embryonic growth. In a stratified analysis for mode of conception, we found negative associations, especially for the pregnancies that were conceived spontaneously.

### 4.2. Strengths and Limitations

The main strengths of our study are the use of longitudinal measures of CRL and EV for embryonic growth from as early as 7 weeks GA. Embryonic growth trajectories were assessed using serial 3D ultrasound, which enabled us to measure trajectories of embryonic growth very precise. To classify neighbourhood deprivation, we chose to use a continuous and frequently updated measure. This allowed us to investigate associations between different levels of neighbourhood deprivation and embryonic growth more precisely than, for instance, using a measure such as binary classification of deprived neighbourhoods. The distribution of NSS in our study population (IQR −1.13–0.77) approximately resembles the distribution of NSS across the Netherlands (IQR −0.57–0.71). The lower limit of the IQR of NSS in our study population indicates the presence of deprived neighbourhoods, which is to be expected in the urban city of Rotterdam [31]. Women from 193 different neighbourhoods were included in the study, which indicates a large neighbourhood heterogeneity within our study population. The demonstrated associations between individual characteristics and NSS are in line with literature, stating that, generally, in deprived neighbourhoods, residents are of non-Western geographic origin, are lower educated and have a higher BMI [6,32]. Therefore, we substantiate the assumption that the NSS is a representative measure for the deprivation status of the particular neighbourhoods [33]. In the interest of comparison, classification of the NSS in three groups (low, intermediate, high) was made.

Some limitations of the present study need to be addressed as well. Possible misclassification may have occurred by neighbourhood SES if women moved during pregnancy to a neighbourhood with a different NSS. However, social mobility in pregnancy is limited and if women move, they generally tend to move within the same neighbourhood or to a neighbourhood with a comparable level of poverty [34,35]. Women with a higher socioeconomic status, and who most often live in neighbourhoods with a higher NSS, are more prone to participate in cohort studies, which introduces selection bias [36]. Moreover, within the eligible population, the included women had a healthier lifestyle (i.e., more frequent adequate intake of fruit, vegetables and folic acid supplements) compared to the women that were excluded from the analysis. As a result, the presented results might be an underestimation of the described associations. Lastly, this study was carried out in a tertiary hospital setting, characterized by the high number of included IVF/ICSI pregnancies. No difference in methodology and quality of the procedure of IVF for women in deprived neighbourhoods compared to IVF for women in non-deprived neighbourhoods is expected, because the costs of the first three IVF cycles are covered by general health care insurances. However, a selected hospital study population may hamper the generalizability of the results to the general population.

### 4.3. Interpretation

Embryonic growth trajectories, expressed as serial CRL and EV measurements, are associated with perinatal outcomes [37]. It is also known that individual risk factors, such as an unhealthy diet and a higher BMI, often accumulate within deprived neighbourhoods [1,6,33,38]. These factors are supposed to underlie the adverse (perinatal) health outcomes that are associated with neighbourhood deprivation, and neighbourhood deprivation operates as a proxy for these individual factors [4,5,7]. To analyse the associations between accumulated individual risk factors as well as the independent effect of living in a deprived neighbourhood with embryonic growth, we studied the associations in models that were only adjusted for GA at the time of ultrasound examination (model 1) as well as a for individual risk factors associated with embryonic growth (model 2). In the fully adjusted models, we found significantly negative associations between neighbourhood deprivation and embryonic growth trajectories, suggesting an independent effect of neighbourhood deprivation on embryonic growth. A neighbourhood effect above and beyond the effect of individual factors, nutrition and lifestyle on pregnancy course and outcome is previously demonstrated in the literature [5,33,39]. This emphasizes the need in clinical practice to consider the total environmental and social background when performing a risk assessment for adverse pregnancy course and outcomes [40,41].

With regard to CRL, which is the most frequently clinically implemented measure, we found a 0.49 mm and 1.08 mm difference between the neighbourhoods with the lowest and highest deprivation score at 7 and 11 weeks GA, respectively. These differences are very small compared to previously investigated associations between other risk factors and embryonic growth. For example, when comparing black, white and Asian women, ethnicity accounts for a 0.81 and 1.28 mm difference in CRL at 6 weeks GA and 1.61 and 2.57 mm difference at 12 weeks, respectively [42]. No or post-conception initiation of folic acid supplement use results in 0.76 mm and 1.63 mm smaller CRLs at 7 and 11 weeks GA, respectively [11]. Finally, a strong adherence to the “high fish and olive oil, low meat” dietary pattern is associated with a 1.9 mm and a 3.4 mm increase in CRL at week 7 and 11 GA, respectively [9].

We expected to find CRL and EV to be smaller in neighbourhoods with a lower NSS, i.e., the deprived neighbourhoods. We found the opposite, namely that in deprived neighbourhoods, embryonic growth trajectories are slightly increased. However, it may be questioned whether the estimate of the increased embryonic growth can be regarded as optimal. As previously studies demonstrated, accelerated growth during early pregnancy increases the risk of excessive birthweight with the associated negative outcomes for mother and infant [43,44]. A proposed underlying mechanism for development of adipose tissue in the first trimester of pregnancy is epigenetic modifications that affect adipocyte development [45,46,47]. It is unlikely that the demonstrated associations are due to measurement errors of the embryonic growth trajectories, as a good intra- and interobserver reproducibility of CRL and EV in early pregnancy has been established [16]. Misclassification due to the method of pregnancy dating cannot be ruled out. After stratification according to mode of conception (and thus pregnancy dating), little differences in the association between NSS and embryonic growth between the groups of spontaneous pregnancies and IVF/ICSI pregnancies were demonstrated. In the end, we adjusted the models for mode of conception (and thus pregnancy dating). At last, we hypothesize that residual confounding of unmeasured intrinsic and extrinsic factors, such as mental stress, might explain the unexpected negative association between the degree of neighbourhood deprivation and embryonic growth. From the literature, it is known that there is an association between living in a deprived neighbourhood and perceived stress [48]. Moreover, chronic stress is involved in the causative pathway of excessive oxidative cellular stress leads that increases cell growth and survival [49]. Excessive oxidative stress is associated with epigenetic hypomethylation of *IGF2 DMR*, amongst other important embryonic growth genes, resulting in an increased expression of growth factors and thus larger embryo’s [8,50,51,52,53,54,55].

## 5. Conclusions

In conclusion, we observed a negative association between neighbourhood deprivation and embryonic growth, above and beyond the effects of individual factors. These rather unexpected negative associations might be explained by stronger unmeasured intrinsic and extrinsic factors, such as mental stressors, contributing to epigenetic hypomethylation and subsequent increased expression of embryonic growth genes. The clinical relevance of the demonstrated small differences in embryonic growth trajectories is unknown. Additional research in larger periconception and birth cohorts in urban regions is warranted in order to further understand the consequences of exposure to deprivation and underlying mechanism in the periconception period on embryonic development, adverse pregnancy outcomes and health and disease risks in later life and future generations.

## Figures and Tables

**Table 1:** Maternal baseline characteristics and nutrition and lifestyle behaviours of the total study population and stratified for neighbourhood status score (NSS) categories.

	Study Population *n* = 566	Low NSS *n* = 170	Intermediate NSS *n* = 280	High NSS *n* = 116	Missing *n* (%)
Neighbourhood status score, median (IQR)	0.02 (−1.13–0.77)	−1.63 (−2.55– −1.14)	0.19 (−0.26–0.57)	1.47 (1.21–1.68)	-
Age (years), median (IQR)	32 (29–36)	33 (30–36)	32 (29–35)	33 (30–36)	14 (2.5)
Nulliparous *n* (%)	244 (46.3)	72 (46.2)	126 (47.7)	46 (43.0)	39 (6.9)
Geographical origin (Western), *n* (%)	475 (87.0)	120 (73.6)	251 (92.6)	104 (92.9)	20 (3.5)
Educational level, *n* (%)					20 (3.5)
High	317 (58.1)	96 (58.9)	134 (49.4)	87 (77.7)	
Intermediate	188 (34.4)	52 (31.9)	116 (42.8)	20 (17.9)	
Low	41 (7.5)	15 (9.2)	21 (7.7)	5 (4.5)	
BMI (kg/m^2^), median (IQR)	24.4 (21.9–28.2)	25.3 (22.7–29.7)	24.4 (21.9–28.3)	23.3 (21.1–26.2)	42 (7.4)
Mode of conception (spontaneous), *n* (%)	332 (58.7)	116 (68.2)	148 (52.9)	68 (58.6)	-
Folic acid supplement use (adequate), *n* (%)	447 (82.6)	124 (77.0)	225 (84.0)	98 (87.5)	25 (4.4)
Fruit intake (adequate), *n* (%)	292 (55.2)	87 (56.9)	146 (54.7)	59 (54.1)	37 (6.5)
Vegetable intake (adequate), *n* (%)	184 (34.8)	64 (41.8)	84 (31.5)	36 (33.0)	437 (6.5)
Alcohol consumption (no), *n* (%)	369 (68.0)	55 (34.2)	86 (31.9)	33 (29.5)	23 (4.1)
Smoking (no), *n* (%)	467 (85.8)	136 (84.0)	238 (88.1)	93 (83.0)	22 (3.9)

BMI, Body mass index; IQR, interquartile range; NSS, neighbourhood status score. Folic acid supplement use: adequate = initiation of folic acid supplement use (400 µg/day) before pregnancy; Fruit intake: adequate = ≥2 pieces/day. Vegetable intake: adequate = ≥200 g/day. Values are percentages for categorical variables, means (SD) for continuous variables with a normal distribution, or medians (25th, 75th percentile, interquartile range) for continuous variables with a skewed distribution.

**Table 2:** Univariate associations between maternal baseline characteristics, nutrition and lifestyle behaviours and the neighbourhood status score (NSS) for the total study population.

Total Study Population (*n* = 569)	β (95% CI)	*p*-Value
Age (years)	0.01 (−0.002, 0.03)	0.09
BMI (kg/m^2^)	−0.05 (−0.06, −0.04)	<0.001 ***
Parity (multiparous vs. nulliparous)	−0.06 (−0.20, 0.07)	0.34
Geographical origin (non-Western vs. Western)	−1.30 (−1.49, −1.10)	<0.001 ***
Educational level (low vs. intermediate)	−0.22 (−0.47, 0.04)	0.10
(high vs. intermediate)	0.29 (0.15, 0.43)	<0.001 ***
Mode of conception (spontaneous vs. IVF/ICSI)	0.37 (0.25, 0.50)	<0.001 ***
Folic acid supplement use (inadequate vs. adequate)	−0.47 (−0.64, −0.29)	<0.001 ***
Fruit intake (inadequate vs. adequate)	−0.001 (−0.13, 0.13)	0.99
Vegetable intake (inadequate vs. adequate)	0.08 (−0.06, 0.21)	0.28
Alcohol consumption (yes vs. no)	0.06 (−0.07, 0.20)	0.38
Smoking (yes vs. no)	0.10 (−0.08, 0.28)	0.29
Total risk score	0.003 (−0.01, 0.02)	0.72

BMI, Body mass index; CI, confidence interval. Folic acid supplement use: inadequate = no initiation of folic acid supplement use (400 µg/day) before pregnancy, adequate = initiation of folic acid supplement use (400 µg/day) before pregnancy. Fruit intake: inadequate = <2 pieces/day, adequate = ≥2 pieces/day. Vegetable intake: inadequate = <200 g/day, adequate = ≥200 g/day. *** *p* < 0.001.

**Table 3:** Associations between neighbourhood status score (NSS) and embryonic growth trajectories, expressed as longitudinal crown–rump length (CRL) and embryonic volume (EV) measurements in the total study population.

Total Study Population *n* = 566	Model 1		Model 2	
	β (95% CI)	*p*-Value	β (95% CI)	*p*-Value
CRL (√mm)	−0.015 (−0.034, 0.003)	0.11	−0.025 (−0.046, −0.003)	0.03 *
EV (^3^√cm^3^)	−0.008 (−0.018, 0.001)	0.10	−0.015 (−0.026, −0.003)	0.01 *

Model 1: adjusted for gestational age only. Model 2: adjusted for maternal age, gestational age, geographic origin, educational level, BMI, mode of conception, folic acid supplement use, fruit and vegetable intake, alcohol use, smoking habits and mode of conception. * *p* < 0.05.

## Supplementary Materials

The following are available online at https://www.mdpi.com/2077-0383/8/11/1913/s1, Figure S1: Full adjusted models for (A) crown-rump length and (B) embryonic volume in the lowest, middle and highest groups of the NSS, Table S1: Maternal baseline characteristics of women who were included and excluded from analysis, Table S2: Maternal baseline characteristics and nutrition and lifestyle behaviours of the study population, stratified for spontaneous pregnancies and IVF/ICSI pregnancies, Table S3: Associations between the neighbourhood status score (NSS) and embryonic growth trajectories, expressed as longitudinal crown-rump length (CRL) and embryonic volume (EV) measurements, stratified for spontaneous pregnancies and IVF/ICSI pregnancies.
