# Supplementary material for: The Impact of Neighbourhood Deprivation on Embryonic Growth Trajectories: Rotterdam Periconception Cohort
Source: J Clin Med. 2019 Nov 8;8(11):1913. doi: 10.3390/jcm8111913 (PMC6912493; doi:10.3390/jcm8111913)
Supplement: Supplementary file 1 [file jcm-08-01913-s001.pdf]

Tables and figures

Figure S1. Full adjusted models for (A) crown-rump length and (B) embryonic volume in the lowest, middle and highest groups of the NSS.

Table S1. Maternal baseline characteristics of the women included and excluded from analysis.

Table S2. Maternal baseline characteristics and nutrition and lifestyle behaviours of the study population, stratified for spontaneous pregnancies and IVF/ICSI pregnancies.

Table S3. Associations between the neighbourhood status score (NSS) and embryonic growth trajectories, expressed as longitudinal crown-rump length (CRL) and embryonic volume (EV) measurements, stratified for spontaneous pregnancies and IVF/ICSI pregnancies.

Figure S1. Full adjusted models for (**A**) crown-rump length and (**B**) embryonic volume in the lowest, middle and highest groups of the NSS.

A

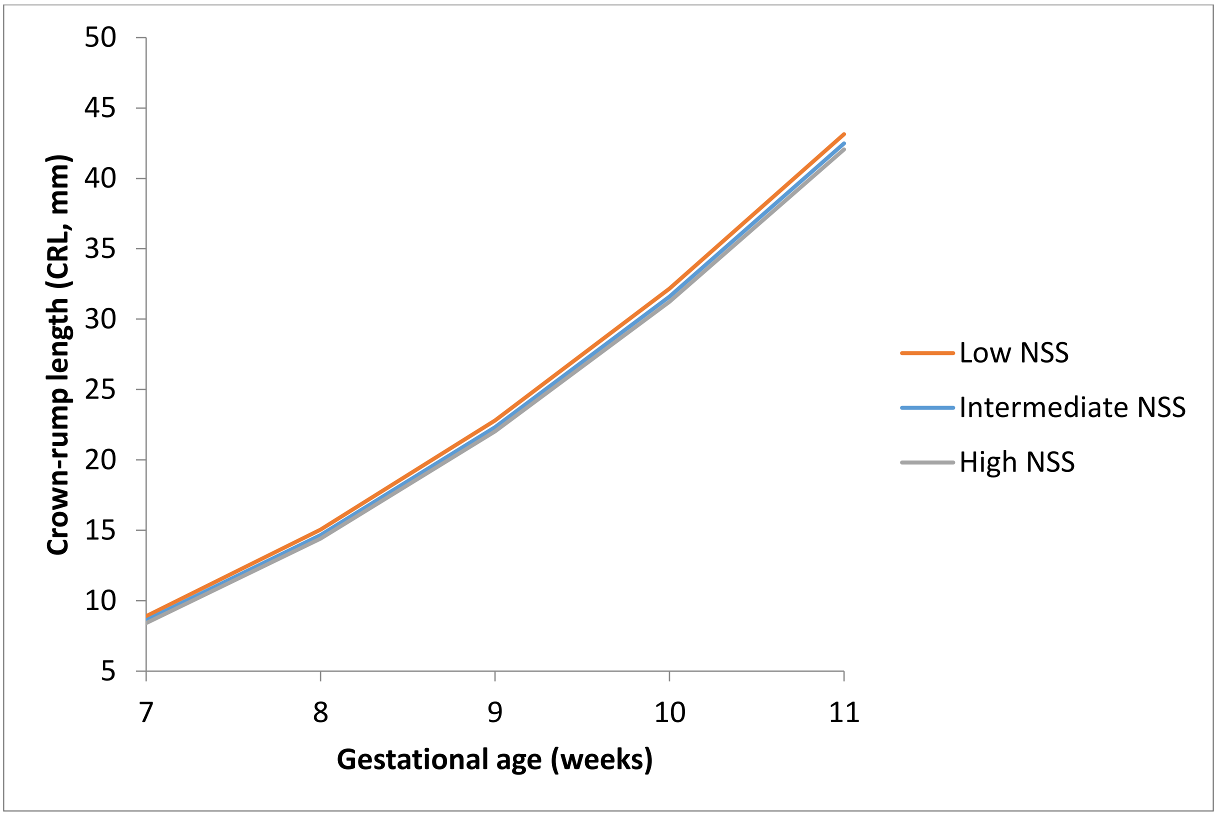

B

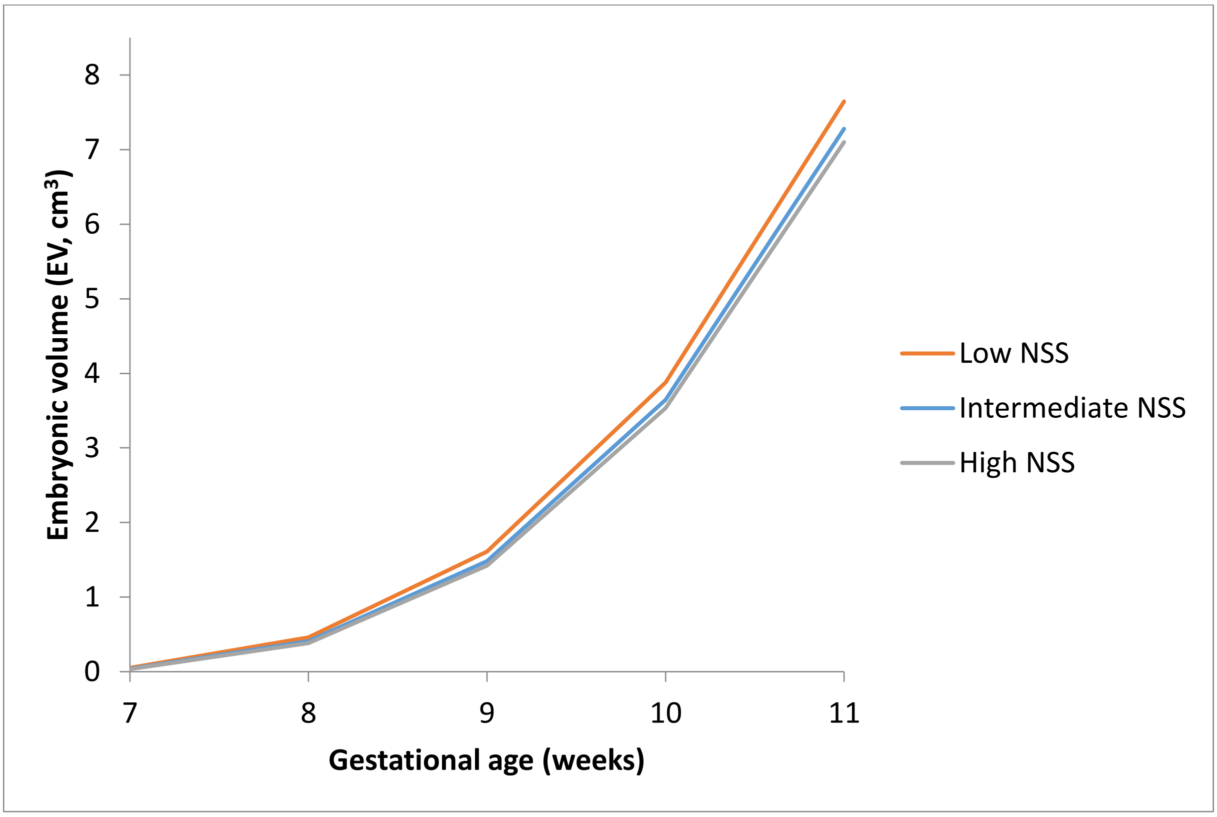

Table S1. Maternal baseline characteristics of women who were included and excluded from analysis.

Characteristics	Included (n = 566)	Excluded (n = 264)	P-value
**Neighbourhood statusscore, median (IQR)**	0.02 (-1.13 – 0.77)	-0.18 (-1.36 – 0.98)	0.37
**Age (years), median (IQR)**	32 (29 – 36)	31 (28 – 34)	<0.001***
**Nulliparous, n (%)**	244 (46.3)	109 (41.3)	0.71
**Geographical origin (Western), n (%)**	475 (87.0)	206 (78.0)	0.18
**Education, n (%)**			0.15
High	317 (58.1)	123 (50.8)	
Intermediate	188 (34.4)	95 (39.3)	
Low	41 (7.5)	24 (9.9)	
**BMI (kg/m^2), median (IQR)**	24.4 (21.9 – 28.2)	24.8 (22.1 – 29.4)	0.19
**Folic acid supplement use (adequate), n (%)**	447 (82.6)	178 (67.4)	0.003**
**Fruit intake (adequate), n (%)**	292 (55.2)	111 (42.0)	0.02*
**Vegetable intake (adequate), n (%)**	184 (34.8)	63 (23.9)	0.02*
**Alcohol consumption (no), n (%)**	369 (68.0)	164 (62.1)	0.25
**Smoking (no), n (%)**	467 (85.8)	198 (75.0)	0.23

BMI, Body mass index; IQR, interquartile range. Folic acid supplement use: adequate = initiation of folic acid supplement use (400 μg/d) before pregnancy; Fruit intake: adequate = ≥2 pieces/day;

Vegetable intake: adequate = ≥200 grams/day. Values are percentages for categorical variables, means (SD) for continuous variables with a normal distribution, or medians (25th, 75th percentile, interquartile range) for continuous variables with a skewed distribution. Statistical testing was carried out through Mann-Whitney U tests and chi-square tests. *$p<0.05$, **$p<0.01$, ***$p<0.001$

Table S2. Maternal baseline characteristics and nutrition and lifestyle behaviours of the study population, stratified for spontaneous pregnancies and IVF/ICSI pregnancies.

Characteristics	Spontaneous (n = 332)	Missing	IVF/ICSI (n = 234)	Missing n (%)	p-value
**Neighbourhood statusscore, median (IQR)**	-0.04 (-1.36 – 0.77)	-	0.08 (-0.84 – 0.77)	-	<0.001***
**Age (years), median (IQR)**	32 (29 – 35)	9 (2.7)	33 (30 – 36)	5 (2.1)	<0.001***
**Nulliparous, n (%)**	107 (34.0)	217 (5.1)	137 (64.6)	22 (9.4)	<0.001***
**Geographical origin (Western), n (%)**	285 (87.7)		190 (86.0)		0.24
**Education, n (%)**		6 (1.8)		14 (6.0)	<0.001***
High	205 (62.9)		112 (50.9)		
Intermediate	94 (28.8)		94 (42.7)		
Low	27 (8.3)		14 (6.4)		
**BMI (kg/m²), median (IQR)**	24.2 (22.0 – 28.8)	21 (6.3)	24.6 (21.7 – 27.8)	21 (9.0)	0.06
**Folic acid supplement use (adequate), n (%)**	234 (72.7)		213 (97.3)	15 (6.4)	<0.001***
**Fruit intake (adequate), n (%)**	166 (53.7)		126 (57.3)	14 (6.0)	0.24
**Vegetable intake (adequate), n (%)**	112 (36.2)		72 (30.8)	14 (6.0)	0.06
**Alcohol consumption (no), n (%)**	195 (60.2)		174 (79.5)	15 (6.4)	<0.001***
**Smoking (no), n (%)**	272 (84.0)		195 (88.6)	16 (6.0)	<0.001***

BMI, Body mass index; IQR, interquartile range. Folic acid supplement use: adequate = initiation of folic acid supplement use (400 μg/d) before pregnancy; Fruit intake: adequate = ≥2 pieces/day; Vegetable intake: adequate = ≥200 grams/day. Values are percentages for categorical variables, means (SD) for continuous variables with a normal distribution, or medians (25th, 75th percentile, interquartile range) for continuous variables with a skewed distribution. Statistical testing was carried out through Mann-Whitney U tests and chi-square tests. ***$p<0.001$

Table S3. Associations between the neighbourhood status score (NSS) and embryonic growth trajectories, expressed as longitudinal crown-rump length (CRL) and embryonic volume (EV) measurements, stratified for spontaneous pregnancies and IVF/ICSI pregnancies.

	**Model 1**				**Model 2**			
**Total study population n = 569**	**Spontaneous n = 332**		**IVF/ICSI n = 234**		**Spontaneous n = 304**		**IVF/ICSI n = 196**	
	β (95% CI)	p-value	β (95% CI)	p-value	β (95% CI)	p-value	β (95% CI)	p-value
**CRL (√mm)**	-0.024 (-0.049 ; 0.002)	0.07	-0.001 (-0.027 ; 0.025)	0.92	-0.031 (-0.059 ; -0.002)	0.04*	-0.010 (-0.043 ; 0.022)	0.53
**EV ($\sqrt[3]{cm^3}$)**	-0.013 (-0.026 ; -0.0003)	0.045*	0.002 (-0.012 ; 0.015)	0.81	-0.020 (-0.034 ; - 0.006)	0.01*	0.001 (-0.017 ; 0.018)	0.95

Model 1: adjusted for gestational age only. Model 2: adjusted for maternal age, gestational age, geographic origin, educational level, BMI, mode of conception, folic acid supplement use, fruit and vegetable intake, alcohol use, smoking habits and mode of conception. *$p<0.05$
